# Allergenicity assessment of novel foods - lessons from immune endotyping of food-allergic patients

**DOI:** 10.3389/fimmu.2026.1843740

**Published:** 2026-06-10

**Authors:** Isabela Assugeni, Melike Gezen, Gül Duman, Naphisabet Wanniang, Françoise Codreanu-Morel, Gabriele Gadermaier, Theresa-Maria Boehm, Annette Kuehn

**Affiliations:** 1 Department of Infection and Immunity, Luxembourg Institute of Health, Esch-sur-Alzette, Luxembourg; 2 Faculty of Science, Technology and Medicine, University of Luxembourg, Esch-sur-Alzette, Luxembourg; 3 Department of Allergology and Immunology, Centre Hospitalier de Luxembourg-Kanner Klinik, Luxembourg, Luxembourg; 4 Department of Biosciences and Medical Biology, Paris Lodron University Salzburg, Salzburg, Austria

**Keywords:** allergenicity assessment, *ex vivo* assays, food allergy, novel food, patient endotyping, risk assessment

## Abstract

Novel foods (NF), defined as foods not commonly consumed in Europe before May 1997, represent a growing area of interest in the pursuit of sustainable and nutritionally valuable diets. Before introduction to the market, however, their safety needs to be clearly demonstrated. Food allergy (FA) represents a significant public health burden and may also extend to NF. Regarding FA risk, the current guidelines for NF safety assessment address possible cross-reactivity with known allergens but offer little direction when allergenic potential is unknown. Conventional approaches such as specific IgE testing, often fail to predict clinically relevant immune responses in humans. *In vitro* platforms based on allergen-stimulated human immune cells allow advanced endotyping of FA patients through molecular and functional immune readouts. They might distinguish between FA, asymptomatic IgE sensitization and healthy states based on immune cell reactivity, via effector cells (e.g. CD63+ basophils), T cells (effector cells expressing high IL-5/IFN-γ ratios; CD154+CD69+ memory, CD137+CD154- regulatory, CD25+OX40+ activated CD4+ T cells) or complex signatures (e.g. Th2 cells, memory Treg cells, activated NK cells; homing marker expression including CCR4, CXCR3). We provide an overview of current immune cell-based stimulation assays allowing to differentiate and grade immune responses in samples from FA, sensitized and healthy individuals, on the basis of multi-omics approaches. Those may help identifying which immune markers are most relevant to measure in future cell line-based approaches, such as gut-on-a-chip assays, which represent an important strategy to assess safety of NF.

## Introduction

### Food allergy: from immune basis to clinics

Food allergy (FA) affects up to 10% of the global population, with increasing prevalence particularly among children (1, 2). In classical FA, the Th2-driven pathway leading to food-specific IgE (sIgE) production is well established (3, 4). Epithelial disruption triggers release of the alarmins IL-25, IL-33, and TSLP, which activate dendritic cells, naïve T cells, and type 2 innate lymphoid cells (ILC2s) (5). IL-33 induces OX40L expression on dendritic cells, promoting differentiation of naïve CD4+ T cells into allergen-specific Th2 cells that secrete IL-4, IL-5, IL-9, and IL-13 (6). Th9 cells may also contribute via IL-9 production (7). ILC2s similarly produce IL-5, IL-9 and IL-13 (5). These cytokines drive IgE class-switch in B cells, resulting in allergen sIgE binding to FcϵRI on mast cells and basophils (5). Upon allergen re-exposure (8), FcϵRI-bound IgE can cross-link, triggering effector cell degranulation and release of symptom-causing mediators (4). Current FA diagnosis relies on clinical history, sIgE tests from skin (SPT) and blood, and in some cases, on oral food challenges (OFC) (1). Common food allergens include milk, egg, seafood, peanut, tree nuts and wheat (2). Many patients have multiple FA (9) and thus clinical practice faces complex reactivity patterns, sometimes triggered by novel foods.

### Novel foods: a topic of growing concern

Novel foods (NF), e.g. chia seeds, mung bean protein concentrate, baobab fruit or yellow mealworm, refer to foods that lacked significant consumption within the EU before May 15th, 1997 (10). NF require regulatory authorization to ensure they are safe for public consumption (11). With the new EU regulation applicable since 2018 (EU 2015/2283), transparency of reported NF numbers has improved, with finally 292 requests for the period 2018-2024 (12). Risk assessment of NF is conducted by the European Food Safety Authority (EFSA) (13). Current guidelines for allergenicity risk assessment rely on a weight-of-evidence approach. For NF derived from allergenic foods, labeling is mandatory for 14 priority allergens, e.g. peanut, soybeans, sesame seeds, lupine (Annex II of Regulation (EU) No 1169/2011). If allergenicity is unknown, NF testing mostly focuses on the risk for potential IgE cross-reactivity in predisposed consumers. Four different tiers address cross-reactivity, ranging from literature search, database sequence comparison in case of a novel protein, sIgE binding assays and human studies (i.e. SPT and OFC) (13, 14). Beyond assessing potential IgE cross-reactivity, *de novo* sensitization is less investigated and understood. A recent review of EU approvals in the period 2018–2023 revealed that an allergenicity risk seemed likely in 50% of the approved NF (15). As no validated predictive methods for *de novo* sensitization are currently available, such information on NF is generally lacking (16, 17).

### Immune endotyping: profiling food-allergic patients

Food-allergic patients exhibit substantial diversity in their clinical reactivity profiles (4, 18, 19). Symptoms can range from mild to severe, occur with varying delays and upon low to high allergen exposure (20–22). Clinical trajectories may also vary over the natural course of the disease (resolution vs. persistence) or during immunotherapy (success vs. failure) (23, 24). Various endotyping approaches, spanning from routine to research methods, approximate the underlying immunological heterogeneity (25, 26).

Food-sIgE patterns, especially molecule-resolved signatures, seize potential severity vs. co- and cross-sensitization patterns (e.g. 2S albumins Ara h 2, Ara h 6 vs. PR-10 protein Ara h 8) (27). Multiplex platforms, covering several hundred allergens, measure molecular spreading of the IgE response. Functional IgE-cross-linking using *in vitro* basophil activation test (BAT) (28) which reveals dose-dependent activation (% of CD63+ basophils), correlates with FA reactivity and severity (28, 29). Studies of other immune cells further enhance patient endotyping. Increased frequencies of type 2-polarized memory B cells expressing IgG1 or IgG4, CD23, and germline ϵ transcripts with highly mutated B cell receptors are found in FA, serving as reservoirs for short-lived IgE-expressing cells and precursors to pathogenic high-affinity IgE-producing cells (26, 30). In peanut allergy, higher frequencies of CD154+ effector cells, mainly Th2A phenotypes among CRTH2+ cells, correlate with reactivity to low-threshold doses (31, 32). Moreover, persistent conventional Th2 (CRTH2-CXCR5-CXCR3-CCR6-CCR4+), Th2A (CRTH2+CCR6-) and Th17-like (CRTH2-CCR6+) populations are linked with FA persistence (31, 33, 34).

Although the assessment of patient variability through immune endotypes has proven useful, whether such immune cell-based profiling can advance our understanding of NF allergenicity was so far not investigated. Here, we provide a comprehensive review of endotyping approaches that capture graded immune responses and discuss their relevance for the assessment of NF.

## Immune endotyping in food allergy: scaling responses

### *In vitro* assays based on sIgE-antibody reactivity

#### sIgE binding

As sIgE testing to NF is part of the risk assessment guidelines and reviewed elsewhere (35, 36), this section is kept short. sIgE antibody tests are important tools in clinical practice (27). sIgE responses are mainly scaled at the level of antibody concentrations and diversity of allergen recognitions. Indeed, sIgE titers are strongly associated with likelihood of clinical reactivity. In peanut allergy, a 95% positive predictive value is sIgE ≥ 35 kU_A_/L at 1 year of age and ≥ 2.1 kU_A_/L at 4 years of age (22, 37, 38). Such predictive sIgE are limited to certain food allergens and currently not available for NF. NF-specific sIgE-levels, in comparison with established food allergens, are worth investigating (39–41). Studies comparing established allergens to NF, including peanut vs. cowpea, shrimp vs. mealworm and cod vs. ray and shark reported differential sIgE-levels that correlated with the level of allergenicity power (**Table 1A**). However, the predictive value of such IgE levels remains limited (42).

**Table 1:** Immune endotyping to scale immune responses of food-allergic patients to foods, including novel foods.

Analysis target	Targeted food(s)	Method; patients (number)	Main findings: differential profiles	Reference
A. Antibody binding assays scaling known vs. new food allergens				
IgE	Peanut/pea vs. cowpea	ELISA; Peanut (N = 13), Legume (N = 14)	Peanut patients: • Peanut vs. cowpea: 67.1 vs. 18.5 kU_A_/L Legume patients: • Pea vs. cowpea: 3.1 vs. 0.6 kU_A_/L	2022 Chentouh et al. (50)
	Peanut, soybean vs. other legumes	Immuno line blot; Food-allergic (N = 106)	Legumes treated vs. untreated: • Peanut: 16% vs. 14.2% • Soybean: 10.4% vs. 8.5% • White lupine: 8.5% vs. 13.2% • Green pea: 4.7% vs. 9.4%	2021 Smits et al. (82)
	Cricket, treated vs. untreated	ELISA; Shrimp (N = 4)	Shrimp tropomyosin vs. differently treated cricket tropomyosins: • OD 0.4 vs. OD 0.15-0.35	2021 Hall et al. (83)
	Shrimp/chicken vs. mealworm	ELISA; Shrimp (N = 20)	Shrimp/chicken vs. mealworm: • 21.7 and 3.5 vs. 19 kU_A_/L	2020 Klueber et al. (53)
	Shrimp vs. Yellow mealworm	Dot-Blot; Shrimp (N = 21)	Shrimp vs. mealworm: • 12/21 strong interactions and 8/21 weak interactions	2019 Barre et al. (52)
	Cod vs. ray/shark	ELISA; Cod (N = 18)	Cod vs. ray/shark: • 11.9 vs. 4.8 kU_A_/L and 6.9 kU_A_/L	2019 Kalic et al. (84)
	Shrimp vs. mealworm	ImmunoCAP; Shrimp (N = 15)	Shrimp vs. mealworm: • 11.5 vs. 1.8 kU_A_/L	2016 Broekman et al. (51)
	Pollen vs. Nangai nut	RAST; Pollen (N = 64)	Birch, grass, mugwort pollen vs. Nangai nut: • 11/64 (17%) positive to Nangai nut • Nangai-sIgE inhibition, IC_50_: ca. 2.5, 2.5–3 vs. 3 BU/ml	2002 Sten et al. (85)
B. Effector cell reactivity assays scaling known vs. new food allergens				
CD63+CD203c+ Basophils	Tropomyosins from molluscs	BAT/flow cytometry; Mollusc (N = 86), Healthy (N = 4)	Tropomyosin from *Haliotis discus* vs. from *Alectryonella plicatula*: • 15.28% vs 24.4% CD63+CD203c+ Basophils	2024 Han et al. (86)
CD63+ Basophils	Peanut/pea vs. cowpea	BAT/flow cytometry; Peanut (N = 13), Legume (N = 14)	Peanut patients: • Cowpea proteins 100-1000x higher concentration to activate basophils (>15% CD63+) than peanut/pea Legume patients: • Cowpea proteins 10-100x higher concentration to activate basophils (>15% CD63+) than peanut/pea	2022 Chentouh et al. (50)
CD63+ Basophils	Cod vs. ray/shark	BAT/flow cytometry; Cod (N = 18)	Cod vs. ray/shark: • 28% vs 3.4% and 3.1% CD63+ Basophils	2020 Kalic et al. (84)
RBL–2H3 cell line	Shrimp/chicken vs. mealworm	Hexosamini-dase assay; Shrimp (N = 20)	Shrimp/mealworm vs. chicken: • RBL, at 100 ng/mL stimulation: median of 10%‐11% for shrimp/mealworm, compared to <0.2% with chicken	2020 Klueber et al. (53)
RBL-SX38 cell line	Shrimp vs. mealworm	Hexosamini-dase assay; Shrimp (N = 6)	Shrimp vs. mealworm: • RBL, at 100 ng/mL stimulation: mealworm induced 15-100% of the response of shrimp	2019 Barre et al. (52)
CD63+ Basophils	Shrimp vs. Mealworm	BAT/flow cytometry; Shrimp (N = 15)	Shrimp vs. mealworm: • 10/15 vs. 11/15 positive in BAT Mealworm raw vs. processed: • Patient-individual differences	2015 and 2016 Broekman et al. (51, 87)
C. Immune cell stimulation assays, readout by flow cytometry - scaling differential immune states				
PBMC	Peanut	Mass cytometry; Peanut (low vs. high threshold, N = 16 and 10)	Allergic patients with reaction vs. without reaction, baseline vs. *in vivo* peanut-exposure in OFC: • Decreased Th2 cells, memory Treg, activated NK cells and increased homing marker expression vs. increased Th2-shifted CD4+ T cells and Treg cells	2023 Klueber et al. (59)
PBMC	Peanut	Flow cytometry; Peanut (N = 58), Healthy (N = 3)	Allergic patients vs. healthy, upon peanut extract stimulation: • Higher frequency of CD154+CD69+ memory CD4+ T cells in early activation	2023 Lozano-Ojalvo (58)
PBMC	Peanut	Flow cytometry, mass cytometry; Peanut active OIT (N = 95); Placebo (N = 25)	Allergic patients under active OIT after treatment vs. baseline, upon peanut extract stimulation: • Reduced release of IL−4, IL−5, IL−9, IL−13 and increased release of MCP−1, MCP−3, IL−12p70 and FLT3L Allergic patients under active OIT vs. placebo, upon peanut extract stimulation: • Reduced frequencies of IL-4+, IL-9+, IL-10+ and OX40+ CD4+ T cells • Increased frequencies of CD27+ and CD38+ CD4+ T cells	2022 Kaushik et al. (61)
PBMC, CD3+ T cell- or CD56+ cell-depleted	Peanut	Flow cytometry/spectral, mass cytometry; Peanut (N = 7), Healthy (N = 7); OIT (N = 6)	Allergic patients vs. healthy, upon peanut extract stimulation: • Increased frequencies of CD86+ NK and B cells, CD56brightCD16–CD69+, CD56dimCD16+CD57–CD69+ and CD56dim CD16+CD57+CD69+ NK cells • Increased expression of IL-4, IL-13, IL-9, IL-10 and IFN-γ in CD69+EM CD4+ T cells	2022 Zhou et al. (62)
PBMC; sorted naïve CD4+ T cells	Peanut, multi-food	FACS, flow cytometry; Peanut (N = 20), Multi-food (N = 20), Healthy (N = 19)	Allergic patients vs. healthy: • Higher proportion of conventional CD11c+ DCs and activated memory-like Tregs Upon anti-CD3/CD28 stimulation: • CD4+ T cells producing lower levels of IL-6, TNF-α and IFN-γ	2021 Neeland et al. (59)
PBMC	Peanut	Flow cytometry; Peanut (N = 33), Healthy (N = 11)	Allergic patients vs. healthy upon anti-CD3/CD28 stimulation: • Higher expression of IL-13 in CD4+ T cells	2020 Wang et al. (88)
PBMC	Peanut	Mass cytometry; Peanut (N = 12), Peanut-sensitized (N = 12), Healthy (N = 12)	Allergic patients vs. sensitized: • Upon PMA/ionomycin stimulation: produced high levels of TNF-α, lower levels of IL-2 in naïve CD4+ T cells • Upon peanut protein solution containing Ara h 1/Ara h 2 stimulation: more peanut-reactive CD4+ T cells with CD45RA- memory phenotype Allergic patients vs. healthy: • Upon PMA/ionomycin stimulation: lower levels of IFN-γ in effector memory CD4+ HLA-DR+ T cells	2020 Neeland et al. (89)
PBMC; *in vitro* expanded T cells	Peanut	Dual ELISPOT assay, Flow cytometry; Peanut (N = 26), Peanut-sensitized (N = 14), Healthy (N = 10)	Allergic patients vs. sensitized, upon peanut antigenic epitopes stimulation: • Higher CRTH2 and lower integrin β7 expression Allergic patients vs. healthy, upon peanut allergen-derived peptides stimulation: • Higher IL-5/IFN-γ ratios	2018 Birrueta et al. (57)
PBMC	Peanut	Flow cytometry; Peanut (N = 17), Healthy (N = 19)	Allergic vs. healthy, upon stimulation: • Higher expression of IL-13, IL-10 by T effector cells Healthy vs. allergic, upon stimulation: • Higher frequency of IFN-γ CD154+ T effector cells	2018 Weissler et al. (56)
D. Immune cell stimulation assays, readout by gene profiling - scaling differential immune states				
PBMC, sorted CD4+ T cells	Peanut	scRNA-seq, TCR-seq/Illumina; Peanut (N = 31), Healthy (N = 5)	Allergic patients under active OIT after treatment, upon peanut extract stimulation: • CD39+ peanut reactive Treg frequency associated with OIT outcome • Lower Th2 signatures (IL4, IL5, IL9, IL13) at baseline correlated with higher peanut tolerated dose after treatment	2025 Han et al. (68)
Whole blood	Peanut	mRNA-seq/Illumina; flow cytometry Peanut (N = 81), Healthy (N = 24)	Allergic patients, baseline vs. *in vivo* peanut-exposure in OFC: • PADI4 and IGF1R gene expression levels were associated with eliciting dose • Neutrophils were negatively associated with peanut threshold	2024 Zhang et al. (66)
PBMC, sorted activated memory CD4+ T cells	Peanut	TCR-seq/immunoSEQ; Peanut (N = 3)	Allergic patients, unstimulated vs. *in vitro* stimulated with peanut extract, 6h vs. 24 h: • CD154+CD69+ memory CD4+ T cells: low TCRβ CDR3 clonotype diversity • CD137+CD154- and CD25+OX40+FoxP3+ Tregs: high TCRβ CDR3 clonotype diversity	2023 Lozano-Ojalvo et al. (58)
PBMC, peanut reactive effector T cells (CD45RA- CD154+ CD4+ T cells)	Peanut	RNA-seq/Illumina; Peanut (N = 16)	Allergic patients, low threshold (CRTH2^+^ pTeff) vs. high threshold dose (CCR6+ pTeff cells) reactivity: • Higher expression of IL17RB, IL1RL1, IL4, IL5, IL13, IL9 and PPARG in CRTH2+ pTeff cells • IFNG, RORC, IL17A, IL17F, IL23R, IL22 enriched in CCR6+ pTeff cells • Central memory differentiation state (CCR7+CD27+) and higher FOXP3 in CCR6+ pTeff cells	2022 Bajzik et al. (31)
PBMC, sorted naïve CD4+ T cells (CD45RA+ CCR7+)	Food allergy	mRNA-seq, DNA methylation/Illumina; Food-allergic (N = 29), Non-food-allergic (N = 18)	Food allergic vs. non-food allergic, upon anti-CD3/CD28 activation: • Lower expression of IFN-γ and IFN-producing cell type gene set • Higher induced Tregs (iTreg) signatures	2022 Imran et al. (70)
PBMC	Peanut, lupin, soy, white bean, green pea	mRNA-seq/Illumina/qPCR; Healthy (N = 10)	Weak (white bean, lupine) vs. strong (soybean, peanut) allergen stimulation: • White bean vs. soy: higher CCL2/CCL7, lower IL-24/RASD2 • Lupine vs. peanut: lower CCL7, higher RASD2	2021 Smits et al. (63)
PBMC	Peanut	DNA methylation/targeted NG BSseq; Peanut (N = 10), Healthy (N = 10)	Allergic patients vs. healthy, upon *in vitro* stimulation with peanut extract: • Differential DNAm signature of genomic regions associated with IL4, IL12B, IL2, IL1B, IL6, BDNF, IL17F, CXCL12, CCR7, RUNX1, CD3ϵ and SERPINE1 genes	2021 Zhou et al. (69)
Whole blood	Peanut	mRNA-seq/Illumina; Peanut (N = 19)	Allergic patients, baseline vs. *in vivo* peanut-exposure in OFC: • Increased expression of NFKBIA, ARG1, CLEC4E, PFKFB2, and ECHDC3 genes associated with severity	2020 Do et al. (65)
PBMC; sorted CD154+ and CD154- T cells	Peanut	RNA-seq/Illumina; Peanut reactive (N = 10), Hyporeactive (N = 10)	Reactive vs. hyporeactive, upon *in vitro* stimulation with peanut: • Higher expression of IL5, IL9, HPGDS, and IL22 and IL26 Hyporeactive vs. reactive, upon *in vitro* stimulation with peanut: • Higher expression of TNFRSF9/CD137 and NFkB inhibitor delta, IL-1 receptor antagonist, vitamin D receptor and CD200	2021 Ruiter et al. (75)
PBMC, sorted CD154+ T cells	Peanut	scRNA-seq/Illumina; Peanut (N = 5), Healthy (N = 4)	Allergic patients vs. healthy, upon *in vitro* stimulation with peanut: • IL4, IL5, IL9, IL13, and IL17RB, IL3 and CSF2 enriched transcriptional state in CD154+ CD4+ T cells	2018 Chiang et al. (67)
Whole blood	Peanut	mRNA sequencing/Illumina; Peanut (N = 19)	Allergic patients, baseline vs. *in vivo* peanut-exposure in OFC: • Increased expression of LTB4R, PADI4, IL1R2, PPP1R3D, KLHL2, ECHDC3 genes associated with clinical reactivity	2017 Watson et al. (64)

BAT, basophil activation test; DC, dendritic cell; ELISA, enzyme-linked immunosorbent assay; EM, effector memory cell; FACS, fluorescence-activated cell sorting; IC_50_, half-maximal inhibitory concentration; mRNA-seq, messenger RNA sequencing; NG BSseq, next generation bisulfite sequencing; NK, natural killer cell; OD, optical density; OFC, oral food challenge; OIT, oral immunotherapy; PBMC, peripheral blood mononuclear cell; PMA, phorbol 12-myristate 13-acetate; RAST, radioallergosorbent test; RBL assay, rat basophil leukemia assay; scRNA-seq, single cell RNA sequencing; TCR-seq, T-cell receptor sequencing; Teff, effector T cell; Treg, regulatory T cell.

Epitope platforms are other powerful tools of antibody endotyping, currently used in research to scale immune responses, including shifts of allergen-specific B cell responses in allergen-specific immunotherapy (36, 43–45). Direct epitope comparisons of established allergens vs. NF are evasive but would be valuable for advanced cross-reactivity testing.

#### Effector cell reactivity

BAT assesses IgE-mediated activation of basophils via cross-linking of FcϵRI-bound IgE, as a functional readout of IgE-mediated hypersensitivity (46). While BAT uses primary cells from fresh patient blood, cell line-based systems require passive sensitization using patients’ sera, followed by *ex vivo* stimulation. These systems include rat basophilic leukemia (RBL) cell models, e.g. 2H3, SX38, and mast cell activation assays (MAT), e.g. LAD2, Hoxb8 (46–48). These assays have demonstrated high sensitivity and specificity, often outperforming sIgE assays and SPT in FA. In this context, studies have shown that BAT and MAT can distinguish between FA and asymptomatic IgE sensitization (48, 49), which may make them promising tools for NF testing. *Ex vivo* effector cell assays rely on stimulation using serial allergen dilutions, in which allergen-induced responses are assessed in a graded manner. This is particularly helpful for comparing clinical groups of patients but also for the comparison of different food proteins or sources (**Table 1B**). In BAT, peanut-allergic and legume-allergic patients required cowpea concentrations that were 100-1000-fold and 10-100-fold higher than those of peanut and pea, respectively, to activate basophils (>15% CD63+) (50). Beyond clinical testing, allergenicity of mealworms was confirmed using BAT and RBL assays, revealing a high effector cell activation capacity, which supported its classification as a novel food allergen (51, 52). Tropomyosin-calibrated RBL cell assays using tropomyosins from shrimp, mealworm and chicken further identified mealworm tropomyosin as a food allergen (53). Although these assays are limited by the need for sera from food-allergic patients as source of polyclonal sIgE, they may still be useful for NF allergenicity evaluation.

### *In vitro* and *in vivo* assays of cellular immune function

#### Conceptual rationale

In **Tables 1C, D**, we describe differential immune cell responses in humans upon food allergen stimulation. Mostly, such studies are not available in the context of ranking foods but rather in categorizing clinical states (e.g. allergic vs. sensitized, low vs. high threshold) (54). As such differential states may also occur in stimulation assays with NF, we highlight the known profiles for subsequent discussion. Most studies focus on peanut allergy, which is a persistent FA that qualifies as a reference allergen and benchmark in risk assessment (55).

#### *In vitro* stimulations followed by flow cytometry

With focus on immune cell functions measured by flow cytometry, several recent studies have explored differences in cellular phenotypes and abundancy, including levels of soluble immune mediators (**Table 1C**). Studies comparing peanut-allergic vs. healthy participants mostly used peripheral blood mononuclear cells (PBMCs) or T cell subtypes, for allergen-specific or non-specific stimulations. Peanut-induced immune signatures distinguished peanut-allergic from healthy individuals through differences in T cell subtypes, effector T cells (Teff) and regulatory T cells (Treg). In allergic individuals, Teff cells expressed the skin-homing molecule, cutaneous lymphocyte antigen (CLA), suggesting activation after exposure through the skin, along with Th2 cytokine production (56). Indeed, peanut-induced Teff cell responses relate to specific cytokine production, such as high IL-5/IFN-γ ratios, which overall confirms Th2 dominated profiles only observed in allergic patients (57). Also, only in peanut-allergic patients, allergen-induced increases of CD154+CD69+ memory CD4+ T cells, including other T cells (CD137+CD154-; CD25+OX40+) were described (58). In addition, Treg signatures separate healthy from peanut-allergic, with allergic patients generally having a higher frequency of activated, memory-like Tregs (CD3+CD4+CD127lowCD25+CD45RA-CCR7-) (59). Comparing peanut-allergic vs. sensitized patients, peanut-induced differences in T cell response magnitude, epitope specificity and phenotype vary. Increased T cell response in symptomatic patients was linked to IL-10 and IL-17 profiles and only secondary to IL-5. Increased levels of CRTH2 and decreased levels of integrin β7, markers of Th2 phenotype and gut homing, were found in allergic patients compared to sensitized individuals (57). Klueber et al. described peanut-induced blood immune signatures in peanut-allergic patients with or without clinical symptoms during OFC. At symptom onset, profiles were characterized by decreased frequencies of Th2 cells, memory Treg cells, activated NK cells, together with increased levels of homing markers (60). In contrast, in the absence of allergic signs, a controlled response was characterized by Th2-shifted CD4+ T cells balanced by Treg cells and lacking homing markers expression (e.g. CCR4, CXCR3) (60).

CD8+ T cells also play a role in differential immune signatures in the context of oral immunotherapy (OIT). Kaushik et al. reported peanut stimulation-induced differences between two clinical outcome groups after OIT, in which CD8+ T cell subsets were decisive (61). These two subgroups, patients with sustained unresponsiveness (SU) and those with desensitization, differed in the prevalence of CD69+CD8+ T cells, and in the distribution of effector memory (CD45RA-CCR7-) vs. naïve (CD45RA+CCR7+) phenotypes. In addition, CD56^dim^CD16+ NK cells were part of the CD8+ related immune signature (61). A recent study highlighted the role of immune cells beyond the T cell compartment in peanut allergy (62). Upon *ex vivo* stimulation, PBMCs from peanut-allergic patients but not healthy controls, showed increased frequencies of NK cell subsets, specifically CD56^bright^CD16-CD69+, CD56^dim^CD16+CD57-CD69+ and CD56^dim^CD16+CD57+CD69+ cells together with the expression of both Th2 (IL-4, IL-13, IL-9) and immunoregulatory cytokines (IL-10 and TGF-ß). Depletion of CD3+ T cells attenuated peanut-induced NK-cell activation, indicating that this response was T cell dependent (62).

#### *In vitro* stimulations followed by gene profiling

Several studies have investigated immune cell functions by performing sequencing analyses of blood cells, either from PBMCs or whole blood, following allergen stimulation, revealing differential immune signatures (**Table 1D**). To date, only one study has reported gene expression patterns upon PBMC stimulation with different legume extracts (63), while other reported studies are more directly aligned with the clinical context.

Transcriptomic analysis of the whole blood compartment after *in vivo* peanut exposure identified key genes associated with acute peanut allergy, including LTB4R, PADI4 and IL1R2, together with other inflammation-related genes (64). In addition, deconvolution-based estimates of leucocyte proportions revealed peanut-induced changes in naïve CD4+ T cells, neutrophils and macrophages. Similarly, in relation to peanut reaction severity (65), transcriptomic and epigenetic analyses of the whole blood identified the key genes NFKBIA and ARG1, as well as key CpG nodes associated with immune response functions, chemotaxis and regulation of macroautophagy. Neutrophil-related functions were confirmed in this study as well (65). Another study addressed *in vivo* exposure profiles in a cohort of more than 100 patients, revealing blood transcriptional signatures (e.g. IGF1R, PADI4) and neutrophil-related features associated with threshold dose reactivity (66). Most studies used PBMC stimulation followed by RNA sequencing (RNA-seq) to characterize graded immune responses. Using single-cell RNA-seq (scRNA-seq) on sorted Treg and CD154+ T cells, Chiang et al. distinguished peanut-allergic from healthy individuals through differential states of peanut-responsive T cells, characterized by distinct proportions of Treg cells, polyclonally activated CD154+ T cells and peanut-activated CD154+ T cells (67). Dominant clusters of correlated genes were identified, including IL4, IL5, IL9, IL13, and IL17RB (67). A recent scRNA-seq study, focusing on peanut OIT outcome groups and peanut-stimulated CD4+ T cells, described two peanut-reactive types: CD4+ Teff cells (CD4+CD154+) and CD4+ Treg cells (CD4+CD137+CD154-), and a non-peanut-reactive type CD4+ T cells (CD4+CD154−CD137-), as well as further sub-clusters (68). Compared with patients with a less successful outcome, patients who achieved SU initially showed decreased frequencies of Th2-related cells and at the end of OIT, increased expression of Th1-related cytotoxic genes, together with increased CD39 expression in peanut-reactive Treg cells (68). Lozano-Ojalvo et al. further confirmed the diversity of peanut-induced T cell population by sequencing the T cell receptor (TCR) β chains and analyzing the complementarity-determining region 3 (CDR3) (58). CD154+CD69+ T cells, early after peanut activation, exhibited lower CDR3 sequence diversity compared with other populations (58). Zhou et al. identified DNA methylation profiles associated with peanut-induced immune differences between peanut-allergic and healthy individuals, including 12 genes related to adaptive (IL4, IL12B, IL2) and innate (IL1B and IL6) immune responses (68). Others applied RNA-seq on PBMCs to identify immune differences between different clinical states. In sorted peanut-reactive Teff cell population, CRTH2 expression correlated with sIgE levels and with the expression of Th2-associated gene transcripts, including IL17RB, IL1RL1, IL4, IL5 and IL13 (31). Using a genome-wide DNA methylation approach in purified naïve CD4+ T cells, key genes related to Th1/Th2 differentiation, including RUNX3, RXRA, NFKB1A and IL4R, were found to play a role in patients with peanut and/or multi-FA (70). Gene expression linked to inflammatory diseases, such as SLFN12, GLI2 and TNFRSF6B, was detected at quiescence and following peanut activation (70).

#### Co-culture assays

Co-culture systems, especially those combining dendritic cells (DCs) and T cells, can be useful for studying food allergenicity, as they capture aspects of allergen uptake, processing, presentation, epithelial barrier effects and recognition by immune cells (71). Not only can different foods be compared within the same system, these models also allow immune responses to be assessed on a graded scale.

In a FA model, Zuurveld et al. combined intestinal epithelial cells with monocyte-derived DCs, followed by co-culture with human naïve CD4+ T cells (72). This approach revealed differential immune responses to two human milk oligosaccharides, as reflected by distinct cytokine expression patterns (72). To scale the immune response after exposure with high vs. low allergenic tropomyosins, cell lines (i.e. Caco-2, HT-29MTX, or HT-29 intestinal epithelial cells) were tropomyosin-stimulated alone or in co-culture of monocyte-derived DCs and HT-29 cells (73). Differential immune readouts identified that only allergenic shrimp tropomyosin, but not chicken tropomyosin, induced epithelial barrier disruption, fostering type 2 mucosal immune activation (73). Furthermore, to study the effects of allergic sensitization via the airways, a co-culture model of THP-1 monocytes and human alveolar cells was used to investigate shrimp tropomyosin together with selected microbial components from bacteria and fungi (74). Compared to individual exposure, combined exposure to tropomyosin with lipoteichoic acid caused an increased protein secretion of CCL20 and TNF, and increased expression of CCL2, CCL20, TNF and IL8 transcripts (74). Ruiter et al. used a co-culture model, which might be useful in studying differential effects of food proteins (75). Peanut-stimulated myeloid dendritic cells (mDCs), producing the enzyme retinaldehyde dehydrogenase 2 (RALDH2), were co-cultured with naïve Th cells, which then showed increased production of IL-5 and expression of gut-homing integrin α4β7 as a measure of peanut-allergic response (75).

## Perspectives

Further innovative assays might allow identifying graded immune readouts in the context of FA. Microfluidic organ-on-a-chip platforms appear interesting to study how food allergens, including NF, interact with gut cells, the immune system and the microbiome. With fewer ethical constraints, these assays also allow immune activation and sensitization pathways to be studied more realistically than in traditional 2D cell cultures (76). Besides their use in inflammatory bowel disease (77), such models are not well established in FA. A recent study compared the effects of the food additive aspartame in different models of epithelial biology and gut immunology, including a Caco‐2 cell-based assay, a gut-on-a-chip and a human induced pluripotent stem cell (iPSC)‐derived intestinal organoid (78). Aspartame induced NF‐κB activation through enhanced oxidative stress, which resulted in proinflammatory cytokine and chemokine release (e.g. CCL20 involved in recruiting CCR6^+^ DCs, Th17 cells, as well as barrier-immune interactions) — a profile regarded to be a risk for gut health (78).

Food allergen-induced crosstalk between the immune system and the gut microbiome might be a key event in allergic inflammation (79). A recent study revealed that commensal strains of the oral cavity, e.g. *Rothia*, *Staphylococcus*, *Streptococcus* and *Veillonella* species, can degrade peanut allergens, thereby reducing IgE-mediated mast cell activation, and influencing peanut allergenicity (80). This confirms the human microbiome as an important player during food digestion, as it is involved in allergen processing and food sensitivity (81).

## Conclusions

**Figure 1:**
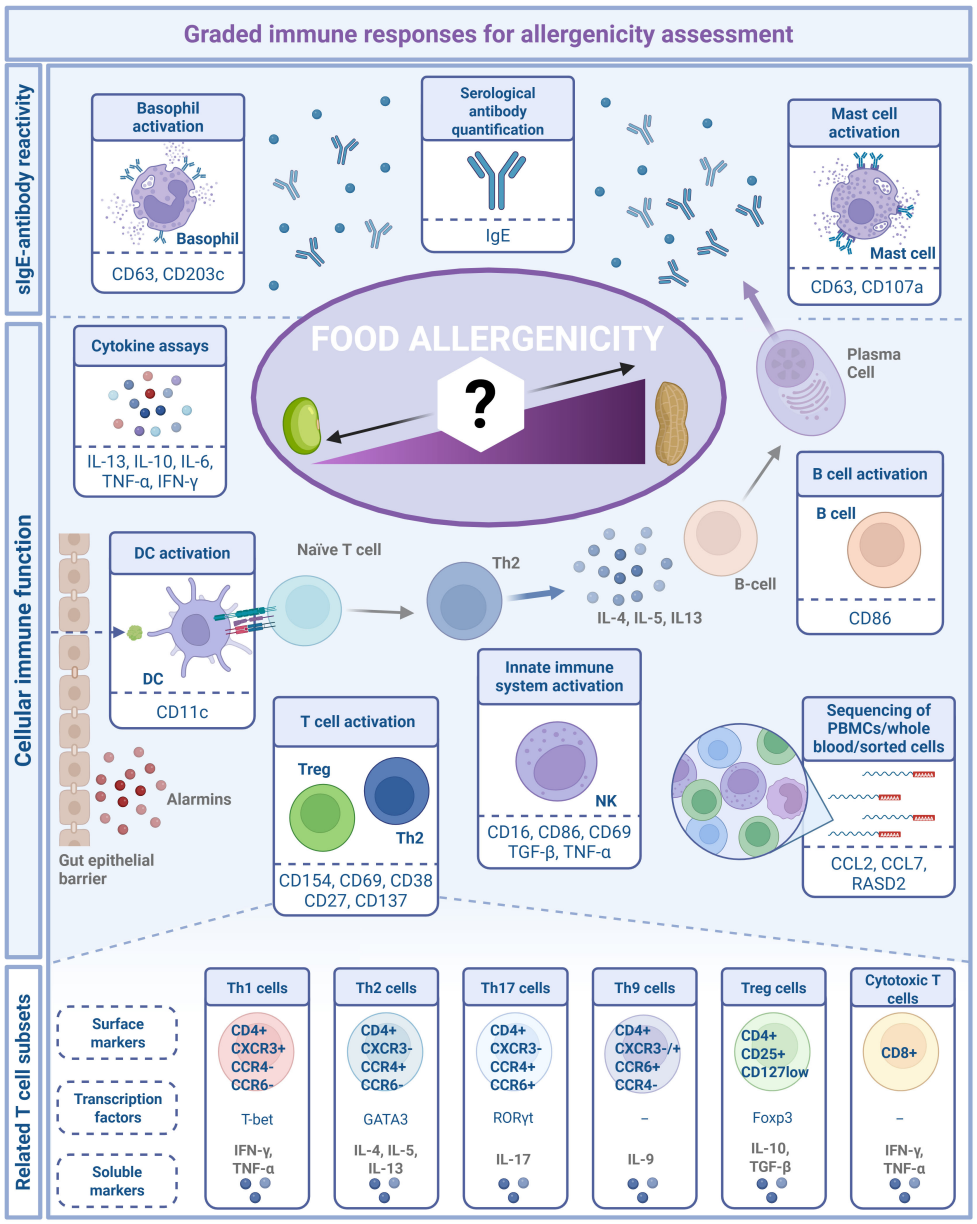
Food allergenicity assessment through grading of immune responses. Parameters for the investigation of immune responses for the distinction of strong vs weak allergenic foods, represented by peanut and mung bean as examples, respectively. The upper panel on sIgE-antibody reactivity comprises serological parameters, including sIgE quantification and BAT. The middle panel on cellular immune function illustrates the promising targets, such as cytokines, reported by *in vivo* and *in vitro* cell-based assays through cytometric and transcriptomic/epigenomic readouts. The lower panel on associated T cell subsets summarizes the main T cell subsets described in stimulation assays, together with their respective surface and soluble markers, and transcription factors. Created in BioRender.com/r1y35kb.

Currently, available tools to assess immune responses to NF, including allergenicity and immunogenicity evaluations, remain limited. Assessing food safety on a risk scale is meaningful because it allows risks to be graded relative to reference foods, such as allergens versus non−allergens. Assays involving sIgE, especially sIgE-mediated effector cells assays (BAT, MAT, RBL), allow such graded immune responses (**Figure 1**, upper panel). Key limitations involve the dependence of BAT on freshly collected blood and, more generally, the necessity for well-characterized and carefully chosen patients’ sera. Cell line-based systems like gut-on-a-chip, mimicking the human immune response including innate and adaptive immune components as well as epithelial structures of physiological relevance, possibly also microbiome influences, would be desirable to test NF in a standardized way. Such systems reflective of graded immune responses in terms of high vs. low reactivity allow proper assessment of NF. Advanced patient-endotyping assays, typically used to characterize patient outcomes and quantify immune responses at different stages, may help to identify which immune markers are most relevant to measure in cell line-based assays (**Figure 1**, middle panel). An integrated approach of human immune cell stimulations and gut-on-a-chip assays might pave the way for amended risk assessment strategies for NF.
